# Cloud-Edge MLOps for Diagnostic Analytics and Anomaly Detection in Smart Office Digital Twins

**DOI:** 10.3390/s26123807

**Published:** 2026-06-15

**Authors:** Saverio Ieva, Davide Loconte, Giuseppe Loseto, Federico Lopomo, Marianna Notarnicola, Andrea Sblendorio, Floriano Scioscia, Michele Ruta

**Affiliations:** 1 Department of Electrical and Information Engineering, Polytechnic University of Bari, via E. Orabona 4, I-70125 Bari, Italydavide.loconte@poliba.it (D.L.);; 2 donkeyPower S.r.l., via E. Orabona 4, I-70125 Bari, Italy; 3 Department of Engineering, LUM “Giuseppe Degennaro” University, S.S. 100 km 18, I-70010 Casamassima, Italy; 4 Lutech S.p.A., Via Orfeo Mazzitelli 258/E, I-70124 Bari, Italyandrea.sblendorio@lutech.it (A.S.)

**Keywords:** Digital Twin, smart buildings, edge computing, Internet of Things, anomaly detection, time-series analysis, MLOps, edge AI, real-time monitoring

## Abstract

Smart buildings require intelligent and scalable solutions to monitor environmental conditions and manage increasingly complex data streams generated by distributed sensing infrastructures. In this context, the paper presents an edge-enabled Digital Twin framework for smart office environments, integrating real-time data acquisition, distributed intelligence, and machine learning-based analytics. The framework adopts a multi-layer architecture composed of a sensor layer, a cloud-edge intelligence layer, and an interaction layer, aligned with Digital Twin reference models. By enabling low-latency processing at the edge and supporting continuous model lifecycle management through Machine Learning Operations (MLOps) practices, the proposed approach overcomes key limitations of traditional cloud-centric solutions. Autoencoder-based models are deployed across the cloud-edge continuum to perform real-time anomaly detection on time-series sensor data. A prototype has been implemented in a real smart office environment, where heterogeneous environmental data are continuously collected and processed. Experimental results demonstrate effective end-to-end data flow, stable long-term operation, and reliable anomaly detection with low-latency response. The system enables real-time monitoring and data-driven analysis of environmental conditions, improving situational awareness and supporting operational decision-making. These findings confirm the effectiveness of integrating Digital Twin technologies with edge AI and MLOps principles for scalable and efficient smart building monitoring systems.

## 1. Introduction

The increasing adoption of Internet of Things (IoT) technologies has become a fundamental enabler for smart building environments, allowing physical spaces to be equipped with networks of smart sensors and actuators capable of continuously observing environmental conditions [1]. In modern smart offices, IoT devices generate large volumes of heterogeneous data streams related to temperature, humidity, air quality, light levels, and energy consumption, creating a detailed digital representation of the building’s operational state. This pervasive sensing capability enables a variety of intelligent services and operational scenarios. For instance, environmental monitoring systems can dynamically regulate heating and air conditioning devices to optimize both energy efficiency and occupant comfort [2,3]. Occupancy and motion sensors can support adaptive space management [4], enabling automatic lighting control or optimizing meeting room utilization [5]. In addition, IoT-enabled monitoring facilitates predictive maintenance by detecting abnormal patterns in equipment behavior before failures occur [6]. However, transforming this continuous flow of data into actionable intelligence remains a significant challenge, particularly when real-time responsiveness and scalability are required. In this context, the Digital Twin (DT) paradigm has emerged as a powerful approach for representing and managing complex Cyber-Physical Systems (CPSs). As highlighted in [7], DT provides a dynamic virtual representation of a physical environment that continuously synchronizes with real-world data, enabling advanced simulation and data-driven monitoring and analysis through the integration of real-time sensing and Artificial Intelligence (AI) models. Beyond the smart building domain, the effectiveness of DTs has been demonstrated in several real-world application scenarios. In the field of energy management, DT have been employed to model the operational behavior of power systems and energy infrastructures [8]. Another promising area concerns sensor network security, where DT can replicate the state and behavior of distributed IoT infrastructures to detect anomalies or malicious activities [9]. Similarly, in logistics and supply chain management, DT have been adopted to mirror the lifecycle of goods, assets, and transportation processes [10].

Nevertheless, traditional DT architectures often rely heavily on centralized cloud infrastructures, which may introduce latency, bandwidth limitations, and potential privacy concerns when processing high-frequency sensor data. To address these limitations, recent research [11,12] has explored the integration of edge computing into DT systems. Edge-enabled architectures allow data processing and Machine Learning (ML) inference to occur closer to the data sources, reducing latency and enabling faster decision-making. This is particularly relevant in smart office environments, where timely detection of abnormal events is critical for maintaining safety and operational continuity.

At the same time, the lifecycle management of ML models deployed in distributed environments remains a complex task. State-of-the-art Machine Learning Operations (MLOps) practices [13] aim to streamline the development, deployment, monitoring, and continuous improvement of ML models through automated pipelines and standardized workflows. Integrating MLOps principles within DT architectures enables continuous learning from real-world data, automated model updates, and robust monitoring of model performance across distributed nodes.

To address these challenges, this paper presents an edge-enabled DT architecture for smart office environments, designed to integrate real-time data acquisition, distributed intelligence, and continuous model lifecycle management within a unified framework. The proposed system combines IoT-based sensing, edge computing, and Deep Learning (DL) models by means of MLOps principles to support near real-time monitoring and anomaly detection, while ensuring scalability and adaptability to evolving environmental conditions.

The main contributions of this work can be summarized as follows:

- The design of a distributed DT architecture integrating sensing, edge intelligence, and cloud-based training components within a unified MLOps framework;
- The implementation of an end-to-end data pipeline supporting real-time data ingestion, preprocessing, and anomaly detection through Autoencoder-based models;
- The deployment of a hybrid cloud-edge infrastructure enabling low-latency inference at the edge and scalable model training within a Kubernetes cluster;
- The integration of a visualization layer providing a 3D DT interface combined with real-time monitoring and anomaly alerting functionalities;
- The experimental validation of the proposed approach in a real smart office environment, demonstrating effective data flow, reliable anomaly detection, and stable edge performance.

The remainder of the paper is organized as follows. Section 2 discusses the background and related work. Section 3 presents the proposed architecture and its main components. Section 4 describes the experimental assessment and evaluates system performance. Finally, Section 6 concludes the paper and outlines future research directions.

## 2. Background

As energy costs increased and available space in cities decreased, it became critical to improve resource consumption efficiency in office spaces [14]. Smart office environments emerged as a response to these concerns. This field encompasses various techniques and technologies, including IoT devices, automated climate control systems, smart workspace furniture, and data analytics tools [15]. The goal is to optimize resource usage, increase productivity, and improve employee health and happiness [16]. This section discusses the key enabling technologies discussed and used in the proposed framework, as well as the issues they seek to address, as well as related works in the field.

### 2.1. Digital Twin and Edge Intelligence for Smart Office Environments

**Figure 1:**
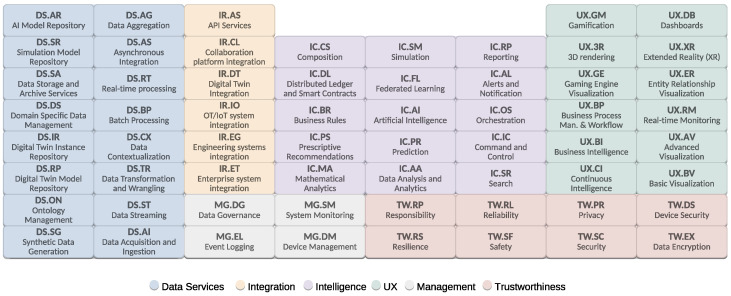
Capabilities Periodic Table [21].

A DT is a class of CPS consisting of three main components: a physical asset in the real world, a high-fidelity multi-scale virtual replica, and a continuous bidirectional data flow between them. DTs enable the monitoring of a product’s entire lifecycle and provide an environment for high-fidelity simulation and analysis [17]. Although this definition is widely accepted, challenges remain in accurately identifying real-world implementations [18]. Systems can be classified as Digital Models (manual data exchange), Digital Shadows (automatic unidirectional data flow from physical to digital), and DTs (automatic bidirectional interaction between two counterparts) [19]. In May 2020, the Object Management Group (OMG) (https://www.omg.org/, accessed on 2 June 2026) established the Digital Twin Consortium (DTC) (https://www.digitaltwinconsortium.org/, accessed on 2 June 2026) with the goal of accelerating the adoption of DT technologies and standardizing the associated glossary. The primary contribution of this organization is the Capabilities Periodic Table (CPT), as illustrated in Figure 1. This framework is architecture- and implementation-agnostic and is intended to decompose and define DTs discrete functional blocks [20]. CPT organizes the functionalities of the DT into six clusters:

- Data Services: It comprises the capabilities required for the end-to-end data lifecycle, including ingestion, cleansing, orchestration, and persistence of high-volume streams originating from the physical asset.
- Integration: It manages the connectivity and interoperability between the digital representation, IoT sensor networks, and legacy enterprise systems.
- Intelligence: This layer represents the computational core and contains physics-based simulations, AI-based analytics, and on-device ML/DL.
- User eXperience (UX): It defines the modalities of human-computer interaction and spans from real-time monitoring dashboards to immersive augmented and virtual reality interfaces for complex data visualization.
- Management: This cluster focuses on the lifecycle management of the DT itself, including provisioning, versioning, system health monitoring, and resource optimization.
- Trustworthiness: This is a critical layer ensuring the security, privacy, resilience, and reliability of the system.

IoT and edge computing are key technologies for developing and implementing DTs. IoT is a field of study that seeks to disseminate everyday objects with communication and computation capabilities, effectively bridging the cyber and real worlds and allowing for sensing and actuation over physical processes and assets [22]. Unlike cloud computing, which shifts all computation to cloud platforms to exploit their resources with benefits in terms of efficiency and up-front cost expenditures, edge computing studies and applies techniques to bring the computation in-situ [23]. These technologies are not mutually exclusive; they can be used in conjunction. Cloud-to-edge architectures can compensate for the shortcomings of a single approach, but they increase the difficulty of system implementation and management. The convergence of cloud-to-edge IoT is usually referred to as cloud-to-thing computing [24]. The term “edge intelligence” in the context of edge computing literature denotes the application of edge computing to AI and ML tasks. Typically, this involves initializing and training a model on cloud computing platforms or capable edge nodes, then forwarding it in a suitable compressed format for inference on lightweight edge nodes or IoT devices [25]. However, recent literature is exploring techniques to perform both training and inference directly on IoT nodes [26]. In DTs, IoT and edge computing provide sensing/actuating technologies, communication capabilities, and computation backbone [23,27].

The DT paradigm finds major applications within the Smart Building and Smart Office domains. In Smart Buildings, DT is closely related to Building Information Modeling (BIM), which is a holistic process of creating and managing all information regarding a built asset throughout its lifecycle based on a 3D model enriched with semantic information. Integrating BIM and DT improves building maintenance and enables real-time monitoring and predictive diagnostics [28]. Other studies utilize DT to enhance the energy efficiency of buildings while maintaining occupant comfort [29], or as a data source for larger-scale urban DTs [30] that supervise smart cities. DT use cases in Smart Office Environments are similar but operate on a smaller scale, such as at the “floor level.” Specifically, they address energy efficiency, occupant preferences, and well-being [31], workers’ health [32], platforms for optimizing office space [33], and workplace safety [34].

### 2.2. Related Work

DTs in smart buildings face significant challenges regarding semantic interoperability between static BIM data and dynamic IoT data streams. The literature addresses this by combining multi-layer architectures with domain ontologies (e.g., ifcOWL [35], SOSA [36], and the Brick schema [37]) and query mediation to integrate heterogeneous data while retaining optimal storage for time-series sensor readings [38]. Other approaches physically extend the Industry Foundation Classes (IFC) schema [39] to include specific operational and maintenance subclasses, using matching tables to link BIM object identifiers directly with external databases [40]. Dynamic BIM, which includes “live” BIM (real-time sensory overlays) and stateful BIM (historical entity tracking), generates contextual features that actively compensate for missing sensor data in centralized systems [41].

In the domain of operational maintenance, DTs are extensively applied to Automated Fault Detection and Diagnostics (AFDD) and predictive maintenance, particularly for Heating, Ventilation, and Air Conditioning (HVAC) systems. Researchers have combined established diagnostic frameworks, such as Air Handling Unit Performance Assessment Rules (APAR), with ML models like Artificial Neural Network (ANN) and Support Vector Machine (SVM) to accurately predict asset degradation and proactively schedule maintenance [41,42]. To manage non-stationary building operations, contextual anomaly detection techniques, such as Bayesian On-line Change Point Detection (BOCPD), have successfully identified anomalous behavioral variations in mechanical assets like centrifugal pumps, effectively filtering out normal operational changes [40]. Semantic frameworks using dynamic BIM features have been shown to significantly improve AFDD diagnostic accuracy, even with limited physical sensor data [41].

Recent studies have also highlighted a shift toward human-centric DTs that support both facility operators and building occupants. For occupants, DTs mapped via 3D scanning provide navigational mobile applications for indoor wayfinding, available space identification, and real-time room booking [43]. For facility operators, integrating IoT motion sensors (such as passive infrared) with DTs and applying machine learning algorithms like tree ensemble learning enables precise, automated forecasting of workspace occupancy. These data-driven space management strategies are highly critical for accommodating modern hybrid working models, directly facilitating proactive space allocation and automated environmental control to substantially reduce heating, lighting, and general energy consumption [44]. Building upon these models, integrating real-time computer vision occupant counts (e.g., YOLOv5) with indoor climate metrics enables adaptive HVAC optimization. Implementing these occupancy-based controls in smart offices reduces ventilation power consumption by 50% [45].

Recent research has also explored the integration of edge intelligence within Human Digital Twin (HDT) environments. In particular, dynamic HDT deployment approaches have investigated two-timescale optimization techniques for latency-aware task execution and resource allocation in end-edge-cloud collaborative environments [46]. Furthermore, federated semantic learning frameworks have been proposed to coordinate distributed AI-generated content and HDT interactions through multi-criteria user selection and privacy-preserving distributed learning mechanisms [47]. Although these studies address important optimization, orchestration, and federated intelligence challenges, their primary focus remains on Human DT scenarios and semantic communication architectures. In contrast, the proposed work focuses on the architectural integration of heterogeneous IoT sensing infrastructures, cloud-edge MLOps pipelines, and real-time anomaly detection within a smart office DT environment, providing an end-to-end operational framework validated through a real deployment scenario.

Despite significant advances in semantic integration, automated fault detection, and occupancy-based optimization, a comprehensive evaluation using the CPT reveals some gaps in existing literature, as summarized in Table 1. Data Aggregation (DS.AG), Data Storage (DS.SA), and Data Transformation (DS.TR) capabilities are well-represented across most studies as a result of the use of ontologies and standard schemas. However, significant limitations remain regarding true real-time execution [38,40,41]. Rather than using event-driven data streams (DS.ST) like Message Queuing Telemetry Transport (MQTT), most current systems process dynamic data through periodic Application Programming Interface (API) polling or retrospective offline dataset analysis [38,45]. Furthermore, although the integration of AI (IC.AI) is becoming standard practice, its application is frequently confined to current-state diagnostics and anomaly detection, rather than true forward-looking forecasting or prediction (IC.PR) [40,41].

The evaluation reveals a distinct lack of focus on the lifecycle management and security of the DT infrastructure itself. Existing frameworks successfully monitor physical building assets (e.g., HVAC power consumption, room occupancy), but typically fail to provide System Monitoring (MG.SM) or Device Management (MG.DM) for the IT/DT nodes hosting the intelligence [42,44]. As a result, there is little literature on Continuous Intelligence (UX.CI), specifically the automated MLOps pipelines required for continuous retraining, deployment, and zero-downtime hot-swapping of ML models. Finally, the architectural Resilience (TW.RS) and advanced Security (TW.SC) protocols are either omitted or only briefly addressed. This work proposes an Cloud-to-edge framework that satisfies specifically these latter CPT dimensions. Is also worth nothing the proposed DT does not yet account for all CPT dimensions. Although MLOps capabilities have been successfully implemented, other dimensions, such as prescriptive analytics (IC.PS), are reserved for future research. Moreover, future research will explore federated learning strategies (IC.FL) to improve system adaptability and protect data privacy (TW.PR) in distributed environments.

## 3. Proposed Architecture

**Figure 2:**
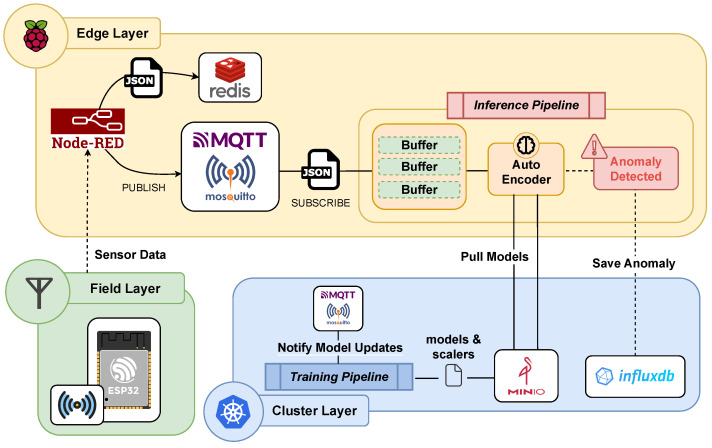
Framework Architecture.

This work proposes an end-to-end distributed MLOps framework for real-time monitoring and anomaly detection in smart building environments. The system adopts a hybrid and distributed computing paradigm, aiming to combine the low-latency capabilities of edge computing with the scalability and reliability of centralized training infrastructures. This framework is designed to handle continuous streams of sensor data and periodically retrain and update ML models. As depicted in Figure 2, the overall network is partitioned into three sections:

- Field Layer. The field layer consists of a network of sensor nodes that are distributed throughout the physical asset, the smart office. As described in Section 3.1, these devices are implemented as ESP32 microcontrollers that acquire environmental data (e.g., temperature, humidity), serialize the readings into JavaScript Object Notation (JSON) [48] payloads, and transmit them to the upper layers.
- Edge Layer. This layer functions as the decision-making engine, providing an end-to-end MLOps framework to automate the lifecycle of ML models. As detailed in Section 3.2, this is achieved through a hybrid computing paradigm that combines centralized computational power with distributed edge resources.
- Cluster Layer. This layer provides computational infrastructure for data storage and model training tasks (Section 3.3). It stores historical sensor data in InfluxDB (version 2.7.6, InfluxData Inc., San Francisco, CA, USA, https://www.influxdata.com/, accessed on 2 June 2026) and periodically trains Autoencoder [49] and Conv1D [50] ML models. Furthermore, it stores and versions model artifacts for later distribution to edge nodes for inference using MinIO (version RELEASE.2025-10-15T17-29-55Z, MinIO, Inc., Redwood City, CA, USA, https://www.min.io/, accessed on 2 June 2026). These components are deployed and orchestrated on a Kubernetes (Version 1.34.0, Cloud Native Computing Foundation, San Francisco, CA, USA, https://kubernetes.io/, accessed on 2 June 2026) cluster.

The devices communicate via MQTT [51] brokers. Messages between the edge node and the field devices are dispatched by the local broker, which is hosted on the edge layer. A second, remote broker is on the cluster layer and handles communication between the edge and the cluster layer. The design philosophy decouples the edge from the cloud, separating computationally intensive model training in the cluster from time-critical inference at the edge. This feature allows the system to update models dynamically without interruption, evolve autonomously by analyzing historical data, and minimize decision latency at the edge. In addition to these, the visualization layer, while not a true layer, provides a human-to-machine interface in the form of a web frontend. Section 4.5 describes the deployment of the web server at the cluster level, which hosts the pages.

### 3.1. The Field Layer: Sensor Network Infrastructure

The Field Layer is the interface between the digital space and the physical environment of the smart building. This layer’s primary function is to acquire raw analog and digital signals, convert them into structured data streams in JSON format for subsequent processing, and transmit them via the MQTT protocol to the upper edge layers, without performing local on-device inference. Field nodes are deployed as ESP32 (Espressif Systems, Shanghai, China) microcontrollers, which were chosen for their optimal balance of computational capability and energy efficiency. These microcontrollers communicate with a wide range of sensors to collect as much environmental data as possible. The adopted sensor suite is composed of the following categories of devices described along with their respective functionalities:

- HVAC and Air Quality Sensing. Internal metrics are captured using integrated DHT11 (Guangzhou Aosong Electronics Co., Ltd., Guangzhou, China, https://asairsensors.com/product/dht11-sensor/, accessed on 2 June 2026) and DHT22 (Guangzhou Aosong Electronics Co., Ltd., Guangzhou, China, https://asairsensors.com/product/am2302-dht22-temperature-and-humidity-sensor/, accessed on 2 June 2026) sensors that measure temperature and relative humidity. These variables are critical for quantifying occupant comfort and are the primary signals used by the system’s anomaly detection models. MQ-2 gas probes (Zhengzhou Winsen Electronics Technology Co., Ltd., Zhengzhou, China, https://www.winsen-sensor.com/sensors/combustible-sensor/mq2.html, accessed on 2 June 2026) also monitor the concentration of combustible gases and smoke, which can be used to build a real-time indoor safety alert system.
- Contextual and Occupancy Sensing: To capture dynamic building utilization, the nodes integrate HC-SR501 Passive Infrared (PIR) sensors (Shenzhen Haiwang Sensor Co., Ltd., Shenzhen, China; https://www.szhaiwang.com/HC-SR501-Pyroelectric-infrared-sensor-human-detector-module-with-10m-distance-pd526372468.html, accessed on 2 June 2026) to detect thermal variations caused by human movement, directly supporting automated lighting control. Light-Dependent Resistor (LDR) modules are used to distinguish between natural and artificial ambient light to manage lighting resources, while normally closed magnetic contact switches monitor the open or closed status of doors and windows to identify perimeter security and potential thermal losses.
- Energy Monitoring: Beyond environmental variables, the field layer utilizes Wi-Fi smart plugs for fine-grained monitoring of electrical loads. These devices capture real-time electrical parameters, including power consumption, voltage, and current, extending the sensing capabilities to include detailed energy efficiency tracking.

The exact spatial distribution and specific network configuration of these nodes within the pilot office environment are detailed in Section 4.1.

### 3.2. Edge Layer: Real-Time Intelligence and Data Management

The Edge Layer’s core components manage field-level data and execute local inference using DL models trained by the Cluster Layer on historical data. The reference device hosting the edge infrastructure is a Raspberry Pi 4 Model B (Raspberry Pi Ltd., Cambridge, UK, https://www.raspberrypi.com/products/raspberry-pi-4-model-b/, accessed on 2 June 2026). The edge layer hosts the local MQTT broker, which is dedicated to dispatch messages from the Field Layer to the edge layer. To communicate with the cluster layer, the edge layer uses the remote broker instead. The software that routes the messages is built on the Node-RED (version 4.0.0, OpenJS Foundation, San Francisco, CA, USA, https://nodered.org/, accessed on 2 June 2026) programming platform. The architecture operates on an event-driven model, with the Node-RED process being invoked at each message.

**Figure 3:**
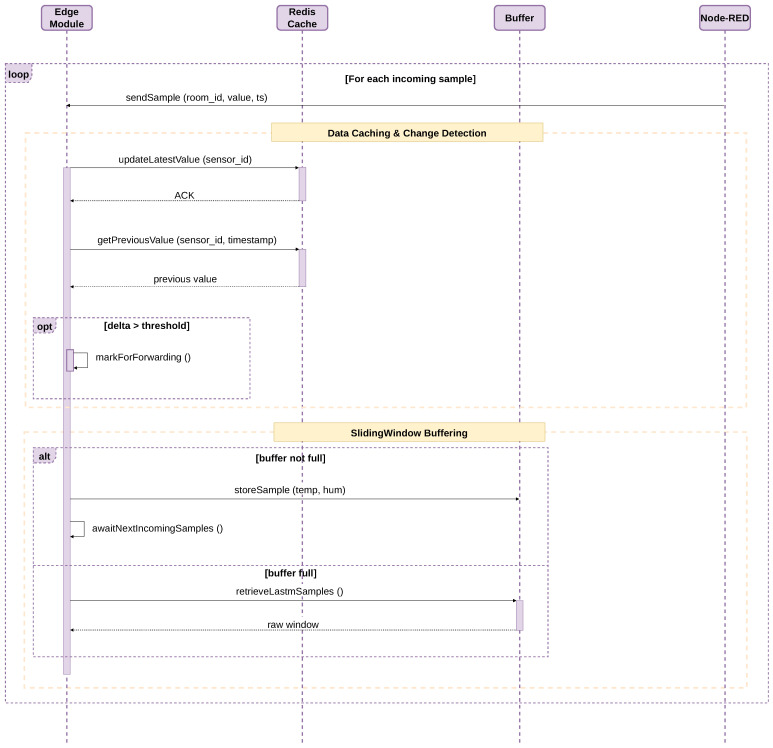
Sequence diagram of the edge-layer data flow: data caching, change detection, and sliding window buffering phases.

Edge layer has several responsibilities. The first one is to apply state-based filtering and buffering, as shown in Figure 3. This optimizes network bandwidth consumption and storage usage. In particular, for each incoming sample, the following steps are executed:

1. The most recent value is updated in the local Redis (version 8.0.2, Redis, San Francisco, CA, USA, https://redis.io/, accessed on 2 June 2026) cache.
2. The previous value for the same sensor is retrieved.
3. If the delta between the current and previous readings exceeds a configurable threshold (Set to 5% of the instrument sensitivity in the current experimental scenario), the sample is marked for forwarding.
4. Concurrently, the sample is sent to a local buffer. This can lead to either of the two following outcomes:
  (a) If the incoming sample does not fill the local buffer, it is stored and the pipeline terminates.
  (b) If enough samples are accumulated capacity, the last *m* are returned to the Edge Module for inference.

**Figure 4:**
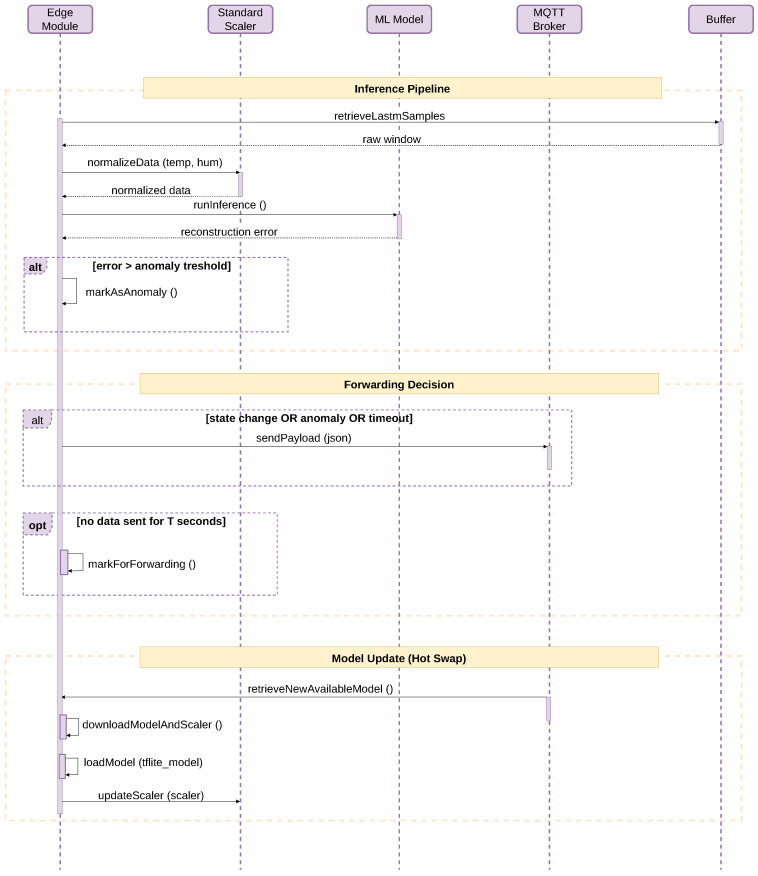
Sequence diagram of the edge-layer diagnostic inference pipeline: normalization, anomaly detection, forwarding decision, and model hot-swap phases.

The Edge Layer holds *n* local buffers, as shown Figure 2, one for each room. In addition to this, the Edge Layer executes the operations depicted in Figure 4. It runs the inference pipeline to process the *m* samples returned from the buffer, normalize the data, and classify anomalies. Anomalies are identified by calculating the reconstruction error [52] from an ANN autoencoder. The model reconstructs the input window and classifies the observation as anomalous if the difference between the output and original data exceeds predefined tolerance thresholds, typically set to 0.1 for HVAC variables. When an anomaly is detected, the pipeline publishes an alert message on a dedicated MQTT topic and marks the data for long-term storage in the cluster layer. The edge layer then forwards this marked data to the remote MQTT broker. Data is also sent if the difference from the previous observation is above a specified threshold or if nothing was transmitted in the last *T* seconds (In these experiments, *T* is set to 60 s). Finally, it subscribes to the remote MQTT broker for model updates, fetching and replacing the local model whenever a notification arrives.

### 3.3. Cluster Layer: MLOps and Infrastructure Governance

The cluster layer consists of a Kubernetes cluster hosting a microservice architecture responsible for updating, versioning, and distributing ML operations, storing historical data, and providing security through Role-Based Access Control (RBAC) policies. Historical data and anomaly detections are stored in InfluxDB, a NoSQL database optimized for large time-series datasets. However, because InfluxDB isn’t suited for storing large binary blobs, updated model checkpoints are kept in MinIO object storage instead.

The proposed architecture features a containerized, automated training pipeline that trains an ANN autoencoder on historical time-series data to generate inference models compatible with the deployment on the edge layer. The neural network is implemented in TensorFlow version 2.20.0 (Google LLC, Mountain View, CA, USA, https://www.tensorflow.org/, accessed on 2 June 2026) and converted to the LiteRT version 2.1.5 (Google LLC, Mountain View, CA, USA, https://ai.google.dev/edge/litert, accessed on 2 June 2026) format for optimization.

**Figure 5:**
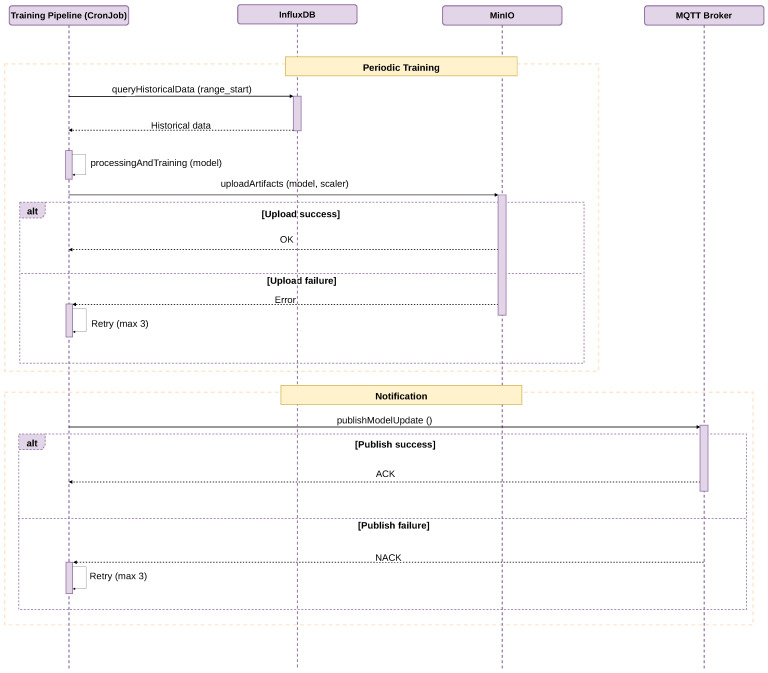
Sequence diagram of the cluster-layer training pipeline: periodic training, artifact upload, and model update notification phases.

The overall process is illustrated in Figure 5. The periodic training job starts with querying historical environmental telemetry from InfluxDB. The pipeline preprocesses these raw measurements through sensor-type filtering, fixed-frequency resampling, interpolation, and outlier removal based on physical validity ranges. In particular, raw sensor data are first filtered by sensor type and target variable (e.g., temperature), and timestamps are converted into a uniform temporal index. The resulting time series are then resampled to a fixed frequency, with missing values handled through linear interpolation, while values outside predefined physical ranges are removed. The processed data are then normalized through standardization, and sliding window techniques are applied to generate fixed-length temporal sequences used for model training. The resulting datasets are split into training and validation subsets while preserving temporal order.

These preprocessing steps are performed within the training pipeline at the cluster level, while the edge layer applies only the corresponding normalization parameters during inference to ensure consistency with the trained models.

Following preprocessing, the data are normalized using a standard scaler and transformed into structured temporal sequences according to a configurable sliding window strategy, allowing the training process to adapt to the temporal dynamics specific to the monitoring scenario. From an algorithmic perspective, the training pipeline relies on autoencoder models, a class of unsupervised learning architectures designed to compress input data into a latent representation and subsequently reconstruct it while minimizing information loss. As explained in Section 3.2, this model can be applied to identify anomalies in the edge layer by using the reconstruction error technique [52]. The trained model and all relevant parameters required for edge execution are converted into a LiteRT-compatible format and stored in MinIO. An MQTT notification is then published to alert the edge devices, prompting the edge layer to download the updated artifacts and instantiate its local inference pipeline. This mechanism enables the automatic propagation of updated models to the edge layer, thereby supporting the continuous feedback loop that underpins the MLOps methodology adopted in the proposed architecture. From a security perspective, access control at the infrastructure level is strictly enforced through a RBAC model, ensuring that each user or service is granted permissions confined to its assigned namespace and operational scope. Authentication and authorization rely on an OpenSSL-based cryptographic workflow. Users accessing the system, typically via secure Secure Shell (SSH) tunnels, generate a private key and a Certificate Signing Request (CSR) that embeds their identity information. This CSR is submitted to the Kubernetes control plane, encapsulated within a resource manifest, and approved by the internal Certification Authority (CA). This design ensures that all certificate signing operations remain internal to the cluster and shielded from external threats. User interactions are subsequently mediated via a kubeconfig file, which binds user credentials and cluster endpoints to enable secure, flexible context switching. The infrastructure is further fortified by Two-Factor Authentication (2FA), stringent password policies, and comprehensive logging mechanisms to ensure full operational traceability. At the application level, identity and access management are centralized through the integration of Keycloak [53]. This open-source platform supports standard authentication protocols, including Open Authorization 2.0 (OAuth 2.0) [54], OpenID Connect (OIDC) [55], and Security Assertion Markup Language (SAML) [56], while providing robust protection against common vulnerabilities such as injection attacks, cross-site scripting, and brute-force access attempts. Furthermore, Keycloak enables fine-grained access control by mapping users to three primary roles tailored to the smart building environment:

- Administrators oversee global system configuration, role assignment, and user management.
- Managers utilize supervisory privileges to configure system parameters, access analytical dashboards, and review historical energy and anomaly data.
- Employees, operating at the field level, are granted restricted permissions focused strictly on real-time monitoring, alarm reception, and maintenance tasks.

## 4. Experimental  Assessment

This section describes the experimental validation of the proposed DT architecture in a real smart office setting. The assessment evaluates the integrated IoT and edge intelligence framework’s effectiveness across multiple dimensions, including deployment feasibility, end-to-end data flow consistency, model lifecycle management, and resource sustainability at the edge. The experimental campaign is designed to showcase the system’s ability to conduct near real-time monitoring, anomaly detection, and continuous learning.

### 4.1. Deployment Scenario

**Figure 6:**
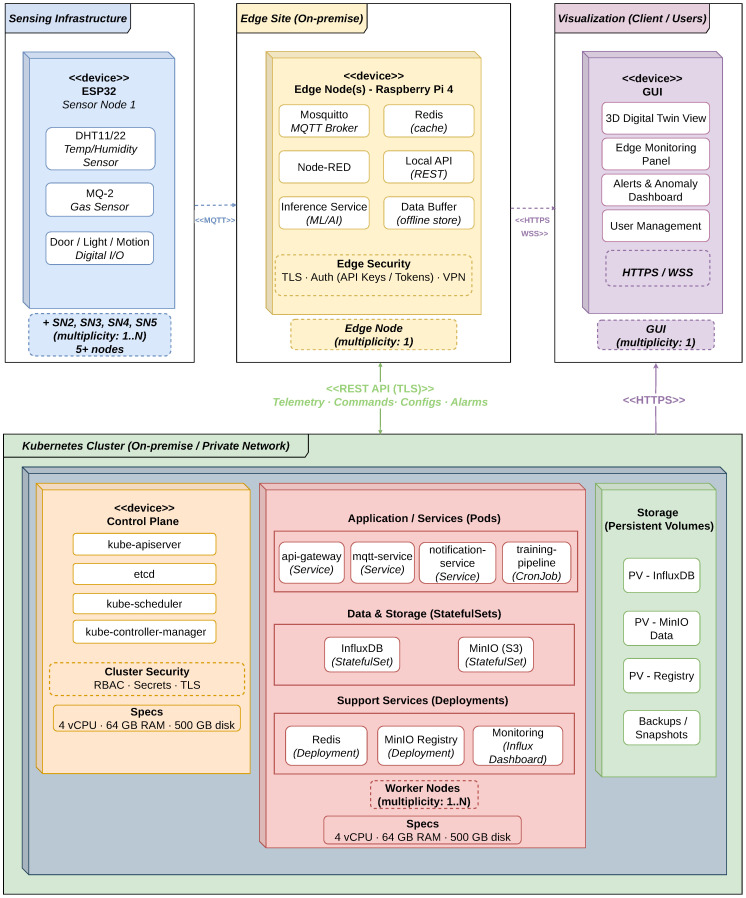
End-to-end deployment of the proposed architecture, showing sensor nodes, edge inference, and cluster-based training.

The experimental validation was conducted through a pilot deployment within a real office environment, specifically in the Living Lab hosted at the premises of Lutech S.p.A. in Bari, Italy, and across multiple strategically selected functional areas. Figure 6 illustrates the concrete end-to-end deployment of the system, highlighting the interaction between sensing devices, edge inference components, and cluster-based training services.

In the experimental deployment, the cloud infrastructure is implemented as a Kubernetes-based cluster consisting of a control-plane node and multiple worker nodes, all of which are provisioned with the same computing resources. Each node is equipped with 4 virtual CPUs, 64 GB of RAM, and a 500 GB virtual disk, all running Ubuntu 22.04 (Canonical Ltd., London, UK). The deployed microservices share the computational resources of the worker nodes. The Kubernetes scheduler manages resource allocation dynamically, assigning pods to available nodes based on CPU, memory, and storage availability. The cluster runs several microservices, such as the MQTT broker, data ingestion components, training pipeline, and model registry using MinIO object storage, as described in Section 3.3.

**Figure 7:**
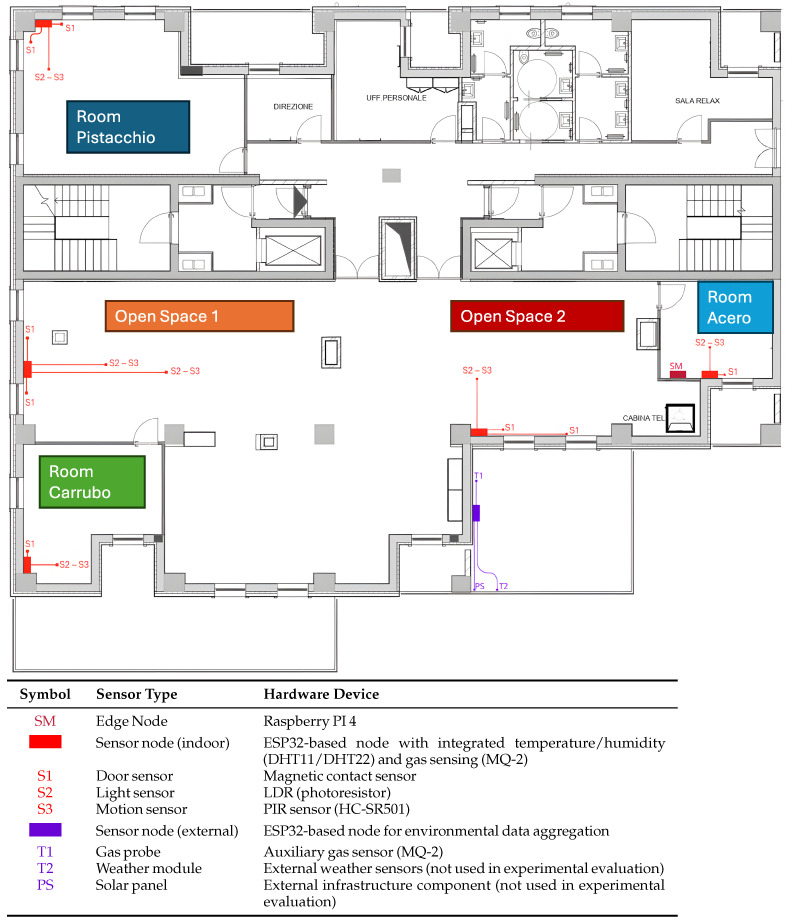
Spatial deployment of the sensor infrastructure and corresponding legend of sensor types and hardware devices.

The floor plan shown in Figure 7 illustrates the spatial deployment of the sensor infrastructure within the reference office environment. The same figure also provides the legend of sensor types and corresponding hardware components.

Anomaly detection is executed locally on a Raspberry Pi 4 that serves as the edge node (SM) as described in Section 3.2. The device is installed in the Sala Acero room and linked to the IoT-Lab Wi-Fi network, which is shared with the ESP32-based sensor nodes, allowing for consistent communication between sensing devices and the edge processing layer. The Raspberry Pi has a 1.5 GHz quad-core ARM Cortex-A72 processor, LPDDR4 memory, integrated Gigabit Ethernet and Wi-Fi 802.11ac connectivity, and USB 3.0 storage for microSD cards or external SSDs. It runs Raspberry Pi OS Trixie 64-bit (Raspberry Pi Ltd, Cambridge, UK) and hosts an open-source software stack, including Node-RED for flow orchestration, Eclipse Mosquitto version 2.0.20 (Eclipse Foundation, Brussels, Belgium) as MQTT broker, and Redis as an in-memory data store for low-latency data access.

The architecture comprises a visualization layer that is logically represented as a separate component in the architecture; however, in the experimental deployment, it is hosted within the cloud infrastructure. The interface interacts with both edge and cluster services through dedicated APIs to provide the visualization services described in Section 4.5. In particular, the visualization module includes a 3D representation of the monitored environment, real-time edge monitoring panels, and anomaly alert dashboards.

Data acquisition is performed at the field layer described in Section 3.1 through multiple ESP32 sensor nodes deployed across the monitored environment. Each sensor node consists of an embedded ESP32 device directly connected to environmental sensing modules, including temperature/humidity and gas sensors, while additional contextual sensors are installed in proximity of the node. These nodes operate as distributed acquisition units and communicate with the Raspberry Pi edge gateway via the IoT-Lab Wi-Fi network using the MQTT protocol. Each node performs local data acquisition and transmits serialized measurements to the edge layer without performing on-device inference, which is instead delegated to the Raspberry Pi.

Sensor nodes are strategically placed throughout the Living Lab, including the Room Acero, Room Carrubo, Rooms Pistacchio, Carrubo, and Open Space areas. This setup ensures coverage of both enclosed environments (e.g., offices and laboratories) and larger shared spaces, enabling evaluation under heterogeneous environmental conditions. The overall sensing infrastructure comprises 28 physical sensors distributed across 6 nodes. Each sensor node (red block) integrates core environmental sensing capabilities. In particular, temperature and humidity sensors are available across all nodes, while gas sensing capability is deployed only in selected environments (Sala Acero and Sala Carrubo). Additional sensing elements, such as door sensors (S1), light sensors (S2), and motion sensors (S3), are physically deployed in proximity to the sensor nodes rather than directly integrated within them. These devices are spatially distributed across the monitored areas but are represented close to the corresponding node in the floor plan for visualization purposes. The deployment also includes additional sensing components, such as smart plugs, auxiliary gas probes (T1), external environmental modules (T2), and solar panels (PS). While these elements are part of the overall testbed and contribute to the heterogeneity of the sensing infrastructure, they are not directly involved in the anomaly detection experiments described in this work. In particular, smart plug data are collected within the system but are not included in the dataset used for model training and evaluation.

### 4.2. Dataset Characterization and End-to-End Data Flow

The dataset used in this study has been generated by the infrastructure described in Section 4.1 and represents the output of a structured aggregation and normalization process orchestrated by the edge gateway. As summarized in Table 2, the resulting dataset comprises a total of 448,124 records collected over a monitoring period of approximately 89 days, corresponding to a data volume of about 81 MB.

The sensing infrastructure comprises 28 physical sensors deployed across multiple environments. However, the final dataset used for the anomaly detection evaluation includes only data streams generated by 15 sensors. The remaining sensors were part of the overall monitoring infrastructure but were not included in the final ML evaluation pipeline. The data collection pipeline is described in Section 3.2. The dataset includes heterogeneous data streams collected from multiple sensor types, as described in Section 3.1. HVAC sensors (DHT11/22) provide temperature (°C) and relative humidity (%) measurements, which constitute the primary signals used for anomaly detection. Power sensors provide electrical parameters such as voltage, current, and power consumption metrics, while additional sensing modalities capture contextual information, including occupancy, door status, and environmental light conditions.

To ensure global uniqueness and avoid identifier collisions across different monitored areas, the system adopts a structured mapping strategy for sensor_id assignment based on the associated node_id. Each physical environment is allocated a predefined identifier range (e.g., Room Pistacchio: 1–10, Acero: 11–20, Room Carrubo: 21–30, Open Space areas: higher ranges). The final identifier is computed as the sum of a base offset associated with the monitored area and a local sensor index.

Initial functional tests successfully validated the end-to-end system’s ability to gather data. A simplified example of the input payload generated by the sensor nodes is reported below:


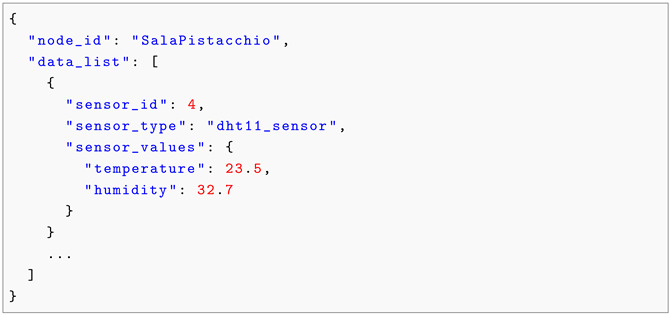


The payload is first transformed into a time-series representation suitable for InfluxDB, where measurements are structured into tags and fields.


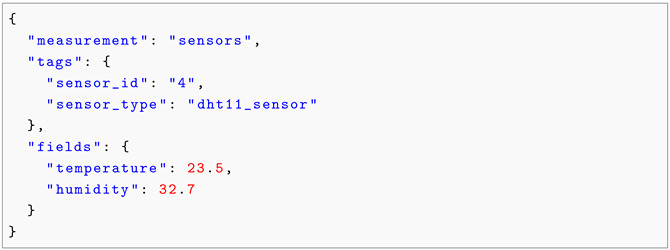


Ultimately, the system was deployed into production over a period of roughly 89 days following validation, which corresponded to a dataset of 448,124 records. The system’s potential for long-term sustainability has been verified by the consistent and reliable propagation of sensor data through the architecture at regular intervals, with a temporal resolution of minutes.

### 4.3. Model Lifecycle Assessment

The model lifecycle assessment evaluates the effectiveness of the integrated MLOps framework, with particular focus on the automation of training, deployment, and updating processes for predictive models, as shown in Figure 5. The system employs a decoupled architecture, which distinguishes computationally intensive training tasks performed within the cluster from low-latency inference operations performed at the edge. This allows the operational model to be continuously updated with new data. The training pipeline described in Section 3.3 is deployed as a scheduled CronJob within the Kubernetes cluster and generates an optimized autoencoder model with each iteration. The process periodically generates deployable.tflite artifacts suitable for edge inference, which include all the relevant parameters, including the model scaler and pre-processing parameters.

**Figure 8:**
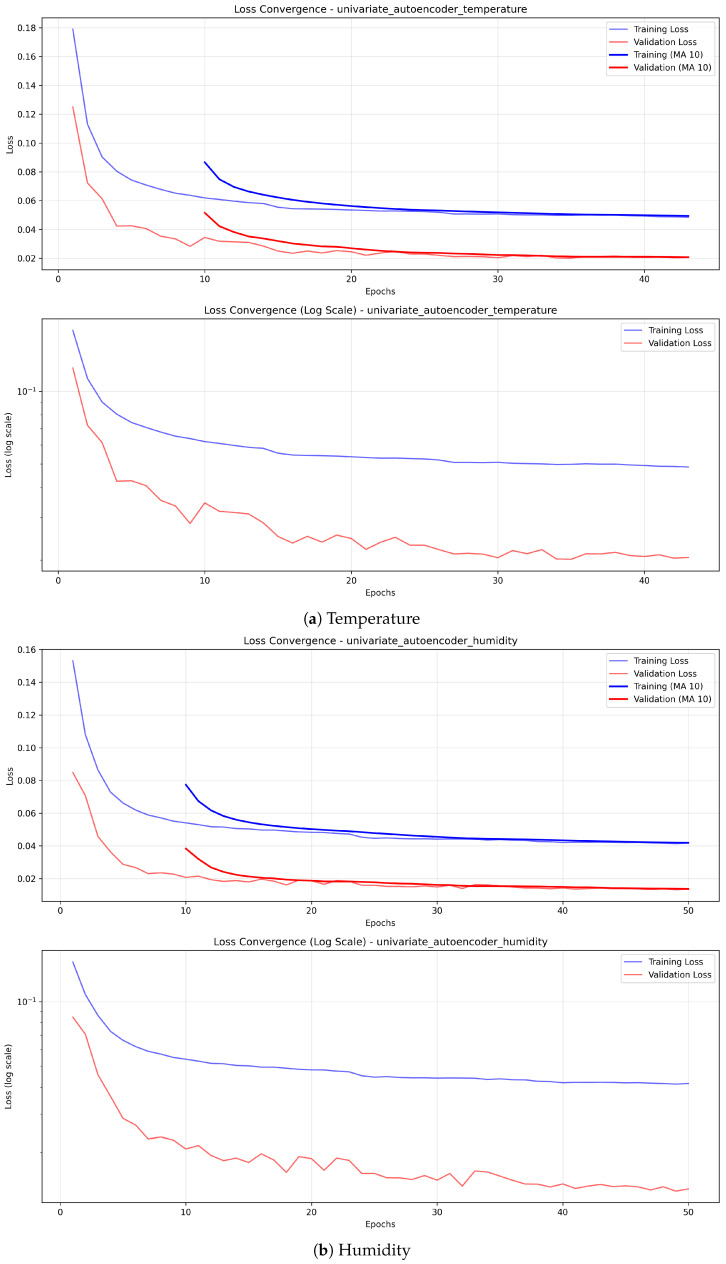
Training curves for the Convolutional Autoencoders.

Currently, the model is trained on a periodic basis from scratch using all the historical data that has been collected thus far. At the edge, the inference service supports dynamic hot-swapping of models: when a new version is notified through the MQTT remote broker, the edge node downloads the updated artifact retrieved from MinIO and loads it without interrupting ongoing monitoring operations. The feedback loop is completed by the edge layer, which stores anomalies detected during inference back into InfluxDB, making them available for subsequent training cycles. Regarding the specific ML task, two univariate anomaly detection tasks were considered for the evaluation: one that detects temperature anomalies and one for humidity series, respectively Temperature AE and Humidity AE. Initially, two architectures have been trained and compared: a baseline Dense Autoencoder and a Convolutional Autoencoder. Subsequent analysis has focused exclusively on the convolutional architecture, as preliminary model selection analysis determined its superior overall performance and convergence speed. The convolutional approach has achieved a 90% reduction in Mean Squared Error (MSE) and has converged 2.5 to 3.3 times faster than the dense network. Nevertheless, this preliminary proof of concept has illustrated that the infrastructure is capable of adapting numerous sensor data streams and heterogeneous neural network architectures. A quantitative comparison between these two architectures is reported in Table 3. Model compilation influences deployment efficiency in terms of model size and resource footprint. As shown in Table 4, the compilation into LiteRT models significantly reduces model size, allowing for efficient deployment on resource-constrained edge devices. The training curves of the models are illustrated in Figure 8.

Anomaly detection is performed using the reconstruction MSE produced by Autoencoder models. The decision threshold is derived from validation data as a percentile of the reconstruction error distribution, typically set to the 95th percentile. Since manually annotated ground-truth anomaly labels were not available in the considered smart office deployment, the evaluation was conducted in an unsupervised setting. Consequently, samples whose reconstruction error exceeded the 95th percentile of the validation error distribution were identified as anomalous. Percentile-based thresholding over reconstruction errors is commonly adopted in Autoencoder-based unsupervised anomaly detection approaches [52,57].

Model validation and promotion are regulated through a multi-stage quality assessment process integrated within the MLOps pipeline. Trained models are evaluated on independent validation datasets using reconstruction-oriented metrics, including MSE, Mean Absolute Error (MAE), and Root Mean Squared Error (RMSE). Reconstruction-error distributions are additionally characterized through statistical descriptors and percentile-based thresholds, which are also used for anomaly identification. Subsequently, newly trained models are compared against the currently deployed baseline model through configurable promotion policies, enabling controlled deployment decisions and preventing performance regressions across retraining cycles. Finally, all evaluated model versions and associated metadata are persistently stored, ensuring full auditability, historical traceability, and rollback capabilities throughout the model lifecycle.

**Figure 9:**
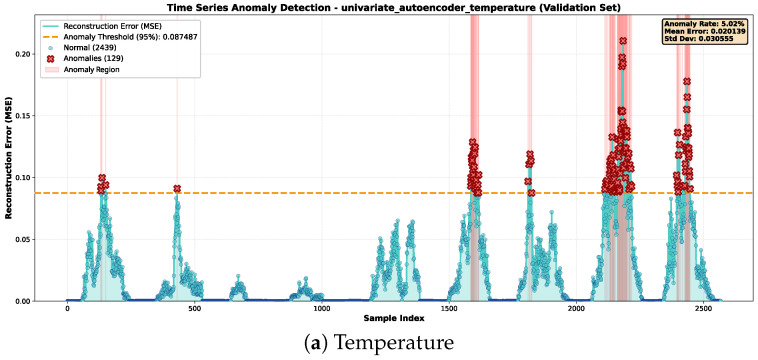

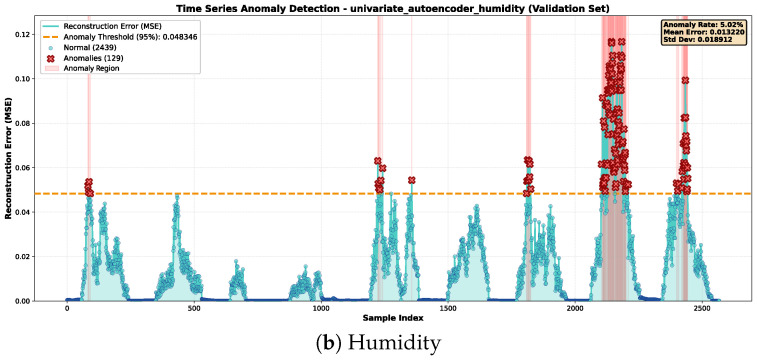
Anomaly detection overlays for the Convolutional Autoencoder.

**Figure 10:**
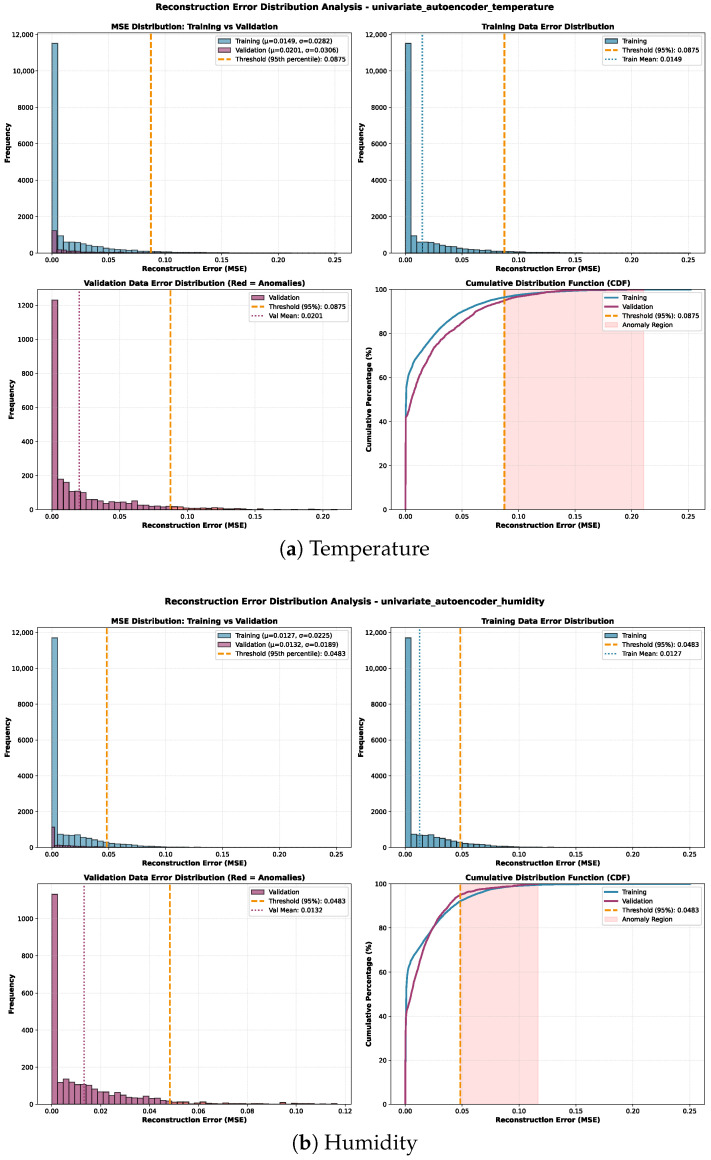
Reconstruction error distributions for the Convolutional Autoencoder.

The effectiveness of anomaly detection is further demonstrated in Figure 9, where anomalous events are clearly identified as peaks in the reconstruction error. The proposed architectures provide a clear separation between normal and anomalous patterns. The distribution of the reconstruction error is reported in Figure 10.

### 4.4. Performance and Edge Resource Assessment

This section evaluates the proposed architecture’s performance in relation to edge deployment constraints, focusing on the Raspberry Pi 4 Model B, which serves as the inference execution gateway. The analysis considers the system’s ability to monitor multiple environments simultaneously while maintaining the responsiveness required for near real-time anomaly detection.

The training parameters are configured with 30 epochs and batch size 32. System responsiveness is primarily influenced by the data acquisition interval and the buffering strategy. Sensor measurements are collected at one-minute intervals and stored in circular buffers. An initial warm-up phase is required to populate the buffer before performing the first inference. Following this phase, the system operates in a steady state, producing anomaly scores with each new acquisition step.

Table 5 report the results of the performance analysis on the edge layer. Experimental measurements on the Raspberry Pi 4 show an average inference time of 42–48 ms per window, with an end-to-end latency ranging between 65 and 120 ms from data acquisition to anomaly detection. CPU utilization remains moderate, averaging 12–15% during normal operation and reaching peaks of 45–55% under concurrent workloads. Memory usage is stable at approximately 185–189 MB during continuous execution, with no evidence of memory leaks. The system demonstrates high reliability, achieving 99.95% uptime and a 100% inference success rate over a continuous 7-day evaluation period. Performance remains consistent across multiple monitored environments, with negligible variance between rooms and stable latency under concurrent multi-room processing.

The proposed architecture enables stable, low-latency anomaly detection on resource-constrained edge devices by deploying models in the LiteRT (.tflite) format, which minimizes memory and computational overhead for real-time execution. Scalability across concurrent multi-room environments is achieved through the MultiRoomDataBuffer, which ensures predictable RAM utilization by maintaining independent, fixed-size windows and processing unitary inference batches. System robustness is further maintained by lightweight background routines, such as periodic health checks, that operate without degrading inference performance. Finally, operational continuity is guaranteed by a dynamic update mechanism that allows new models and data scalers to be deployed without causing operational downtime.

### 4.5. Digital Twin Visualization and Operational Use Cases

The proposed DT’s user interface is provided by the Visualization and Interaction Layer, which allows for real-time monitoring and interactive exploration of the building environment. The system enables operators to inspect system conditions, analyze environmental parameters, and respond to detected anomalies in real time by integrating live data streams within the user interface.

#### 4.5.1. Virtual Representation of the Building

**Figure 11:**
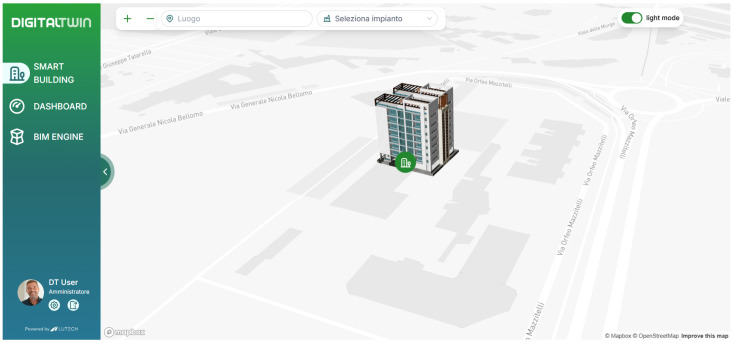
Georeferenced DT visualization integrating the BIM model within a map-based interface.

The visualization layer integrates the BIM-based model with a georeferenced map to create a three-dimensional representation of the building, as illustrated in Figure 11. This virtual environment allows users to explore the monitored spaces and access localized information.

**Figure 12:**
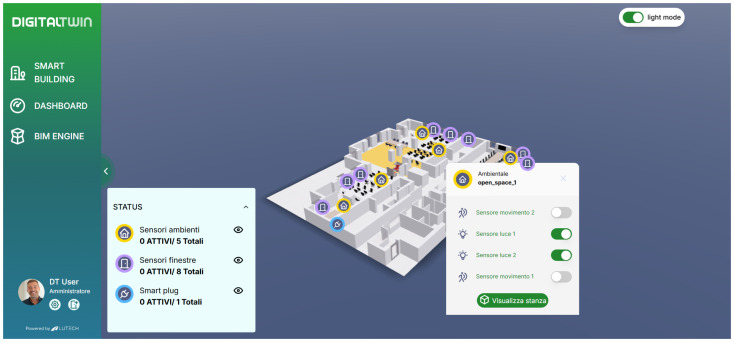
Interactive indoor DT visualization.

Each area of the building is annotated with real-time sensor data, visually overlaid onto the corresponding spatial elements and environmental measurements, such as temperature and humidity, are directly associated with their physical location. As shown in Figure 12, the system allows for detailed indoor navigation using interactive markers to represent sensor nodes. These elements enable users to check the status of individual devices and access room-level information via dedicated interface panels such as contextual dashboards and dynamic pop-up views.

The interface also supports advanced interaction capabilities, such as exploring system components, retrieving detailed information, and interacting with contextual services via an integrated virtual assistant. From a technological perspective, the visualization layer is implemented as a web-based application built on React (Version 19.1.1, Meta Platforms, Inc., Menlo Park, CA, USA; https://github.com/facebook/react, accessed on 2 June 2026) and Next.js (Version 14.2.3, Vercel Inc., San Francisco, CA, USA; https://github.com/vercel/next.js, accessed on 2 June 2026), leveraging Three.js (Version r164, Ricardo Cabello, Barcelona, Spain; https://github.com/mrdoob/three.js, accessed on 2 June 2026) and WebGL (Version 2.0, Khronos Group, Beaverton, OR, USA; https://www.khronos.org/webgl/, accessed on 2 June 2026) for 3D rendering, and Mapbox GL JS (Version 3.4.0, Mapbox, Washington, DC, USA; https://github.com/mapbox/mapbox-gl-js, accessed on 2 June 2026) for geospatial visualization. The system also stores a representation of the building state and allows historical conditions to be reconstructed and analyzed, thus supporting retrospective investigations and comparative analyses over time.

#### 4.5.2. Monitoring and Alarm Management

**Figure 13:**
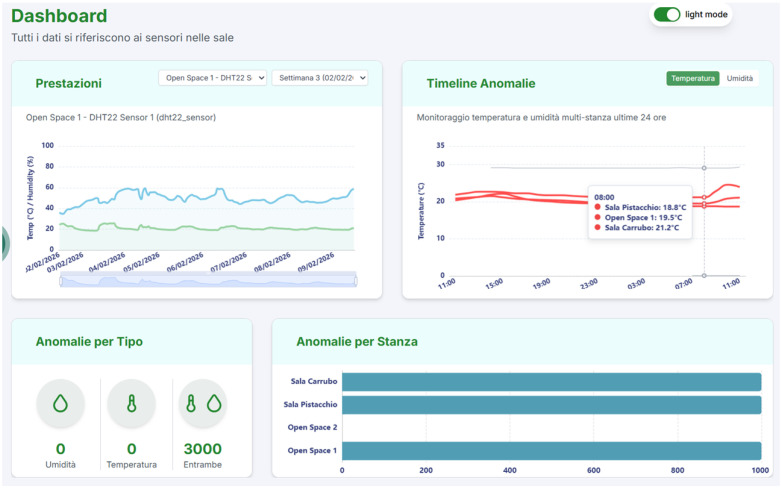
Operational dashboard for real-time monitoring and anomaly visualization within the DT.

The monitoring subsystem provides a comprehensive view of system behavior through dedicated dashboards, as shown in Figure 13. These interfaces allow users to visualize anomalies in real time and quickly identify abnormal conditions.

Anomalies are represented by visual indicators such as highlighted signals and aggregated counters, which are integrated into temporal charts and summary panels. Time-series visualizations enable users to observe changes in environmental parameters, whereas anomaly timelines provide a view of detected events across monitored areas. The dashboard also provides aggregated views, such as categorization by anomaly type (e.g., temperature and humidity) and spatial distribution across rooms.

**Figure 14:**
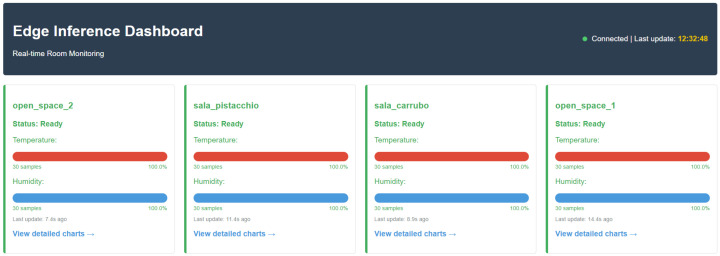
Edge-level dashboard for monitoring inference processes and buffer states.

A lightweight monitoring interface is also deployed directly on the edge gateway. This local dashboard supports debugging and system observability at the edge level by offering real-time inspection of buffer states and inference outputs, as displayed in Figure 14.

#### 4.5.3. Dashboard Use Cases: Energy Monitoring and Predictive Maintenance

The visualization layer extends beyond real-time monitoring. Anomalies are detected and stored over time to create a structured historical record of abnormal events. This historical perspective enables the detection of repeating patterns and progressive deviations in system behavior across multiple monitored environments. These insights are visualized through aggregated views within the dashboard, allowing anomaly trends and frequency distributions to be analyzed across rooms and sensor types. This allows operators to detect critical conditions and predict potential problems. The system also supports data export via dedicated endpoints, allowing for detailed offline analysis of anomaly logs and maintenance planning activities.

Energy monitoring is another relevant use case. Monitoring and analyzing resource consumption in the DT environment can enhance energy efficiency. The system combines data from smart plugs and environmental sensors to provide a detailed view of energy consumption across different areas and devices. Electrical parameters such as power, voltage, and current allow for the analysis of device-level consumption patterns. Environmental sensors, such as light-dependent resistors, enable the system to correlate artificial lighting usage with natural light conditions, assisting in the detection of inefficient behaviors such as excessive energy consumption in well-lit areas. These insights are presented to users in the form of dashboard components that aggregate consumption metrics by room, device type, and time interval, allowing them to identify consumption patterns and potential inefficiencies. Although energy-related data are part of the overall monitoring infrastructure, they are not directly used in the anomaly detection models examined in this paper. Their integration within the DT enables the exploration of energy optimization strategies and supports future extensions toward data-driven energy management.

## 5. Discussion

This section analyzes how the proposed DT architecture satisfies the capabilities defined in the CPT, as summarized in Table 1. The discussion highlights the correspondence between the system components and the CPT dimensions, referencing the sections where each capability has been implemented and validated.

Data Services (DS). The proposed system provides comprehensive support for data aggregation, transformation, and storage. Data ingestion and normalization pipelines described in Section 3.2 and Section 4.2 enable structured acquisition of heterogeneous sensor data, while transformation and preprocessing steps are implemented within the training pipeline (Section 3.3). Time-series persistence is handled by InfluxDB, and real-time data streaming is achieved through MQTT-based communication across system layers.

Integration (IR). The architecture ensures seamless integration between IoT devices, edge components, and cloud infrastructure. As described in Section 3.1 and Section 3.2, sensor nodes communicate through MQTT brokers, while interoperability between distributed services is enabled through API-based communication and message-driven workflows. It can be pointed out how the primary contribution of the proposed work lies in the architectural integration of heterogeneous cloud-edge DT components within a unified operational framework, rather than the introduction of specific ML algorithms or anomaly detection models. In particular, the proposed system combines distributed IoT sensing infrastructures, real-time edge inference, automated MLOps lifecycle management, cloud-edge orchestration, and continuous deployment mechanisms into a single end-to-end architecture validated in a real smart office scenario. From the perspective of the CPT, the novelty of the proposed approach emerges from the simultaneous integration and operational validation of multiple CPT dimensions that are typically addressed independently in the existing literature.

Intelligence (IC). The system implements AI-based analytics through Autoencoder models for anomaly detection, as detailed in Section 4.3. Currently, diagnostic capabilities are realized through univariate time-series anomaly detection using Autoencoder-based predictive models operating on environmental variables such as temperature and humidity. This implementation primarily focuses on validating the operational feasibility and long-term stability of the proposed cloud-to-edge infrastructure in a real deployment scenario, rather than on large-scale benchmarking or advanced multivariate predictive analytics. Continuous retraining pipelines in the cluster layer (Section 3.3) enable periodic model updates based on newly collected data. However, the present MLOps workflow still relies on periodic full retraining and does not yet include online learning, incremental adaptation, or explicit model degradation monitoring mechanisms. Similarly, anomaly thresholds are currently derived from statistical reconstruction-error distributions, with no fully adaptive thresholding policies across different deployment scenarios. Consequently, fully multivariate collaborative reasoning, adaptive learning strategies, and prescriptive intelligence capabilities remain important directions for future research.

User eXperience (UX). The visualization and interaction layer (Section 4.5) provides comprehensive monitoring and analysis tools, including 3D DT visualization, real-time dashboards, and anomaly inspection interfaces. These components support diagnostic and predictive monitoring, enhancing situational awareness and enabling interactive exploration of system data. The integration of real-time monitoring with periodic model updates also enables continuous intelligence capabilities.

Management (MG). Lifecycle management of models and infrastructure is supported through the MLOps pipeline (Section 3.3), which enables automated training, deployment, versioning, and hot-swapping of models. System monitoring and performance evaluation are further validated in Section 4.4. In addition, device-level management functionalities are supported through the monitoring and control of edge components within the infrastructure.

Trustworthiness (TW). Security and access control mechanisms are implemented through RBAC policies and Keycloak-based identity management, as described in Section 3.3. These components ensure secure communication, controlled access to system resources, and protection against common vulnerabilities. The architecture also incorporates resilience mechanisms through distributed deployment and fault-tolerant data processing across edge and cluster layers.

The experimental validation was conducted within a single operational smart office deployment and was primarily designed to evaluate the feasibility, architectural integration, deployment continuity, and long-term operational stability of the proposed cloud-to-edge DT infrastructure under realistic conditions. In this respect, the continuous 89-day deployment naturally exposed the system to sensor variability, environmental noise, heterogeneous device behavior, and transient network instabilities, effectively acting as a long-term operational stress test for the proposed architecture. Nonetheless, large-scale comparative benchmarking campaigns, controlled ablation studies, and broader multi-environment validation scenarios are important areas for future research. While descriptive and diagnostic functionalities are well supported, extending to fully predictive and prescriptive analytics represents an important future research direction.

## 6. Conclusions

This paper presented an edge-enabled DT architecture for smart office environments, designed to integrate real-time data acquisition, distributed intelligence, and machine learning-based analytics within a unified MLOps framework. The proposed system combines IoT-based sensing, edge computing, and cloud-based model lifecycle management to support continuous monitoring, diagnostics and anomaly detection in a scalable and efficient manner. The experimental validation, conducted in a real office deployment, demonstrated the effectiveness of the proposed approach across multiple dimensions. The system ensures a consistent end-to-end data flow, from sensor data acquisition to visualization within the DT, while supporting heterogeneous data storage through both time-series and graph-based representations. The integration of edge-based inference enables near real-time anomaly detection, reducing latency and ensuring timely response to abnormal conditions. From an analytical perspective, the evaluation of Autoencoder-based models confirmed the effectiveness of reconstruction-error-based anomaly detection.

The proposed architecture also demonstrated robustness and scalability in edge deployment scenarios. The use of lightweight models optimized in LiteRT format, together with efficient buffering strategies and dynamic model updates, enables stable operation under resource-constrained conditions. The system supports concurrent monitoring across multiple environments and ensures operational continuity through zero-downtime model updates.

Overall, the results highlight the effectiveness of integrating DT technologies with edge AI to enable scalable and intelligent monitoring systems for smart buildings. The current implementation supports descriptive and diagnostic monitoring capabilities, while laying the foundation for more advanced decision-support functionalities. Future work will cover additional CPT modules by extending the architecture. In particular, this includes the adoption of multivariate and cross-domain models, the integration of additional sensing modalities, and the development of advanced decision-making mechanisms for automated and proactive system management. Further investigations will also explore federated and on-device learning strategies to improve adaptability while preserving data privacy in distributed environments.

## Figures and Tables

**Table 1:** Comparison of DT Frameworks for Smart Buildings (**✓**: supported, **✗**: not supported. Red text indicates a brief explanation of the main divergences w.r.t the current work. Yellow cells indicate partial support based on the currently reported implementation.).

Feature	CPT	[38]	[40]	[42]	[43]	[44]	[41]	[45]	[46,47]	This Work
Data Aggregation	DS.AG	**✓**	**✓**	**✓**	**✓**	**✓**	**✓**	Dataset	**✓**	**✓**
Batch Processing	DS.BP	**✗**	**✗**	**✓**	Reports	**✓**	**✓**	**✓**	FSL	**✓**
Real-Time Processing	DS.RT	Queries	**✓**	Periodic	10-min	**✓**	**✓**	**✓**	**✓**	**✓**
Data Storage	DS.SA	**✓**	**✓**	**✓**	**✓**	**✓**	**✓**	Excel	**✗**	**✓**
Data Streaming	DS.ST	HTTP	BMS	API	10-min	**✓**	**✓**	Offline	Edge	**✓**
Data Transformation	DS.TR	**✓**	**✓**	**✓**	**✓**	**✓**	**✓**	**✓**	**✓**	**✓**
IoT System Integration	IR.IO	**✓**	**✓**	**✓**	**✓**	**✓**	**✓**	**✓**	Wearables	**✓**
Artificial Intelligence	IC.AI	Future	**✓**	**✓**	Future	**✓**	**✓**	**✓**	**✓**	**✓**
Prediction	IC.PR	**✗**	Anomaly	**✓**	**✗**	**✓**	Diagn.	Control	**✓**	**✓**
Dashboards	UX.DB	**✓**	**✓**	**✓**	**✓**	**✓**	Graphs	Plots	**✗**	**✓**
3D Rendering	UX.3R	**✓**	**✓**	**✓**	**✓**	**✓**	**✓**	**✗**	**✗**	**✓**
Continuous Intelligence	UX.CI	**✗**	**✗**	Retrain	**✗**	**✗**	**✗**	**✗**	**✓**	**✓**
Device Management	MG.DM	**✗**	**✗**	**✗**	**✗**	**✗**	**✗**	**✗**	**✗**	**✓**
System Monitoring	MG.SM	Building	Building	Building	Building	Building	Building	Energy	HDT	**✓**
Resilience	TW.RS	**✗**	**✗**	**✗**	**✗**	**✗**	**✗**	**✗**	Optimization	**✓**
Security	TW.SC	**✗**	**✗**	**✗**	Privacy	**✗**	**✗**	**✗**	Privacy	**✓**

**Table 2:** Summary of dataset characteristics.

Metric	Value
Total records	448,124
Observation period	89 days
Data size	81 MB
Number of sensors	15
Sensor types	7
Minimum Sampling interval	1 min ^1^

^1^ Due to the nature of the data ingestion pipeline, 1 min is the maximum period between two successive samples. See Section 3.2 for further details.

**Table 3:** Comparison between Dense and Convolutional Autoencoder performance.

Metric	Dense AE	Convolutional AE
Validation MSE (Temperature)	0.20	0.02
Validation MSE (Humidity)	0.12	0.01
Anomaly Threshold (Temp)	0.71	0.09
Anomaly Threshold (Humidity)	0.46	0.05
Convergence Speed (epochs)	50	15–20

**Table 4:** Model size comparison before and after TensorFlow Lite conversion.

Model	Keras Size (MB)	LiteRT Size (MB)
Temperature AE	0.36	0.04
Humidity AE	0.36	0.04
Compression ratio	∼9x	
Size reduction	∼89%	

**Table 5:** Operational parameters of the edge inference pipeline.

Parameter	Value
Sampling Interval	1 min
Window Size (Univariate)	30 samples
Warm-up Phase	30–60 min
Inference Trigger	Per new data sample
Inference Time	42–48 ms
End-to-End Latency	65–120 ms
CPU Usage (avg)	12–15%
Memory Usage	185–189 MB

## Data Availability

Data available on request due to restrictions (e.g., privacy, legal or ethical reasons).

## Acronyms

2FA	Two-Factor Authentication
AFDD	Automated Fault Detection and Diagnostics
AI	Artificial Intelligence
ANN	Artificial Neural Network
APAR	Air Handling Unit Performance Assessment Rules
API	Application Programming Interface
BIM	Building Information Modeling
BOCPD	Bayesian On-line Change Point Detection
CA	Certification Authority
CPS	Cyber-Physical System
CPT	Capabilities Periodic Table
CSR	Certificate Signing Request
DL	Deep Learning
DT	Digital Twin
DTC	Digital Twin Consortium
HDT	Human Digital Twin
HVAC	Heating, Ventilation, and Air Conditioning
IFC	Industry Foundation Classes
IoT	Internet of Things
JSON	JavaScript Object Notation
LDR	Light-Dependent Resistor
MAE	Mean Absolute Error
ML	Machine Learning
MLOps	Machine Learning Operations
MQTT	Message Queuing Telemetry Transport
MSE	Mean Squared Error
OAuth 2.0	Open Authorization 2.0
OIDC	OpenID Connect
OMG	Object Management Group
PIR	Passive Infrared
RBAC	Role-Based Access Control
RMSE	Root Mean Squared Error
SAML	Security Assertion Markup Language
SSH	Secure Shell
SVM	Support Vector Machine
UX	User eXperience
